# Literary Note

**Published:** 1907-12

**Authors:** 


### LITERARY NOTE.

J. H. Chambers & Co., Publishers, St. Louis, Missouri. Visiting and pocket reference book (perpetual), for the year 1908. It has been revised and enlarged, is handsomely vellum bound, with lapel, price 50c. Convenient to carry in the pocket; containing 128 printed and blank pages. The publishers will mail copy postpaid on receipt of 24-2c. stamps.

